# Neural Competitive Queuing of Ordinal Structure Underlies Skilled Sequential Action

**DOI:** 10.1016/j.neuron.2019.01.018

**Published:** 2019-03-20

**Authors:** Katja Kornysheva, Daniel Bush, Sofie S. Meyer, Anna Sadnicka, Gareth Barnes, Neil Burgess

**Affiliations:** 1 School of Psychology and Bangor Imaging Unit, Bangor University, Bangor, Wales LL57 2AS, UK; 2 Institute of Cognitive Neuroscience, University College London, London WC1N 3AZ, UK; 3 Queen Square Institute of Neurology, University College London, London WC1N 3BG, UK; 4 Motor Control and Disorders Group, St George’s University of London, London SW17 0RE, UK; 5 Sobell Department for Motor Neuroscience and Movement Disorders, University College London, London WC1N 3BG, UK; 6 Wellcome Trust Centre for Human Neuroimaging, University College London, London WC1N 3AR, UK

## Abstract

Fluent retrieval and execution of movement sequences is essential for daily activities, but the neural mechanisms underlying sequence planning remain elusive. Here participants learned finger press sequences with different orders and timings and reproduced them in a magneto-encephalography (MEG) scanner. We classified the MEG patterns for each press in the sequence and examined pattern dynamics during preparation and production. Our results demonstrate the “competitive queuing” (CQ) of upcoming action representations, extending previous computational and non-human primate recording studies to non-invasive measures in humans. In addition, we show that CQ reflects an ordinal template that generalizes across specific motor actions at each position. Finally, we demonstrate that CQ predicts participants’ production accuracy and originates from parahippocampal and cerebellar sources. These results suggest that the brain learns and controls multiple sequences by flexibly combining representations of specific actions and interval timing with high-level, parallel representations of sequence position.

## Highlights

• Non-invasive recordings in humans show “competitive queuing” of upcoming actions
• Queuing gradient mainly reflects a high-level template of sequence position
• Queuing gradient originates in ipsilateral parahippocampal and cerebellar areas
• Strength of queuing gradient predicts production accuracy

## Teaser

Kornysheva et al. use non-invasive magneto-encephalography in humans to decode the preparation of upcoming actions in a sequence. They reveal that the brain preactivates a template of sequence order and that this template predicts the accuracy of subsequent execution.

## Introduction

Most skilled human behaviors evolve in temporally structured sequences. A breakdown of their fluency (e.g., in stuttering, dyspraxia, and occupational dystonia) can profoundly impair everyday functioning (Miller, 1988, Sadnicka et al., 2018, Stein et al., 1996). Historically, two opposing theories have been proposed for the neural basis of motor sequence control. The associative chaining account, originating from the pioneering work of Ebbinghaus in 1885 (Ebbinghaus, 2013), postulated strong forward connections between successive elements of a sequence, leading to the behaviorist idea that each sequence element serves as a conditioning stimulus for the subsequent element during the formation of complex behaviors (Terrace, 2005). In modern neuroscience, this hypothesis has engendered the formulation of state-space models of skilled motor control, such as writing and Morse code production (Goudar and Buonomano, 2018, Laje and Buonomano, 2013, Shenoy et al., 2013, Sussillo et al., 2015). These models are characterized by a spatio-temporal trajectory determined by the serial evolution of population activity, so that a population state *n* triggers the state *n+1*, the latter *n+2*, etc. In neocortex-inspired recurrent neural networks (RNNs), the evolution of multiunit activity is primarily determined by the connectivity matrix acquired through learning. In other words, motor sequences are controlled by a serial transition through neural population states, which are mapped onto motor actuators.

**Figure 1:**
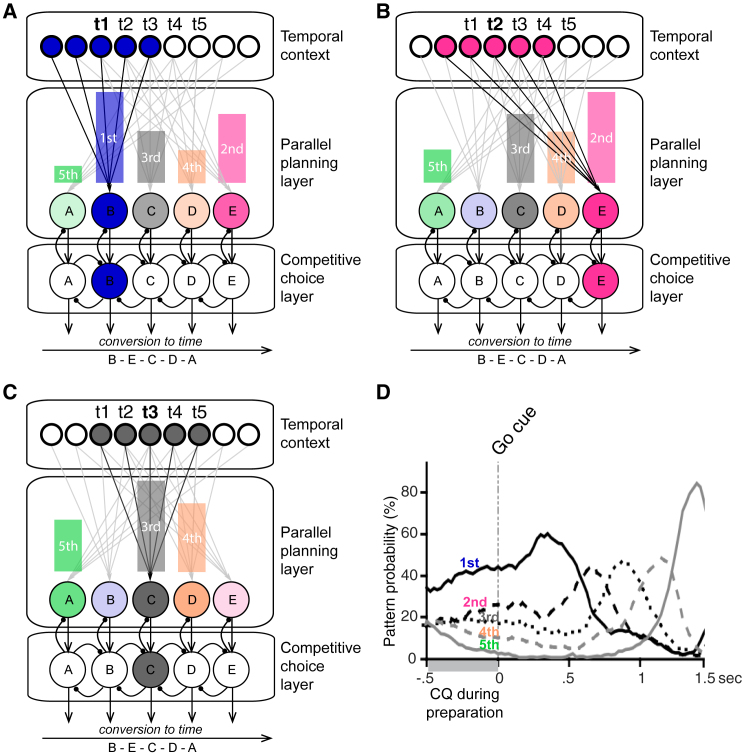
CQ Model and Prior Data (A–C) The Parallel planning and Competitive choice layers of the competitive queuing (CQ) model contains nodes representing possible sequence items, such as finger presses A, B, C, D, and E. When learning a sequence, connections are formed from sequentially activated nodes in the Temporal context layer to item nodes in the parallel planning layer as each is activated in turn. Nodes in the Competitive choice layer receive one-to-one input from corresponding nodes in the parallel planning layer and compete via lateral inhibition so that the most active inhibits the other nodes in its layer, drives motor output, and then inhibits its input from corresponding parallel planning node. Sequence planning is implemented by reactivating the first state in the temporal context layer, producing strong activation of the first item in the parallel planning layer and progressively weaker activation of the following items because of the progressive decrease in overlap between the connections to those items and the current activity pattern in the temporal context layer (A). The most active node in the competitive choice layer wins the competition, generating the corresponding action, and is then self-inhibited through the planning layer, allowing the next most strongly activated node to generate the next action, as in positions two (B) and three (C). This iterative process allows conversion of a parallel planning code into a temporally structured serial output. (D) Averbeck et al. (2002) recorded multi-unit activity in prefrontal cortex while monkeys drew geometrical shapes. The results were consistent with the graded parallel preparation of sequential shape segments, as described by the CQ model above. Reproduced with permission; Copyright (2002) National Academy of Sciences, U.S.A.

However, since Lashley’s seminal proposal (Lashley, 1951), there has been an alternative account, suggesting that all elements of a planned sequence are active simultaneously before execution, leading to the characteristic finding of transposition errors among nearby elements (Rhodes et al., 2004); e.g., as observed in speech or typing. So-called “competitive queuing” (CQ) models can formally explain this behavior by introducing a parallel preparation layer that determines serial order by competitive interactions between sequence elements driven by differing levels of excitation according to the sequence (Figures 1A–1C; see Bullock, 2004 for a review). The most active node wins the competition, generates the corresponding action, and is then self-inhibited through the planning layer, allowing the next most strongly activated node to generate the next action. This process effectively allows a conversion of a parallel planning code into a serial output during execution. Crucially, the respective excitation gradients are learned by associations from a temporal context layer to each sequence element. The pattern of activity in this layer evolves over time during encoding, allowing different items to be associated with different states, and is reset prior to sequence production such that it evolves in the same way as during encoding.

The form of activity in the temporal context layer can be as simple as a decaying start signal (Page and Norris, 1998), a combination of start and end signals (Hartley and Houghton, 1996), or, to capture effects of temporal grouping or rhythm, a sequence of overlapping states (Burgess and Hitch, 1999, Burgess and Hitch, 2006). Importantly, the temporal context layer represents sequential timing or position independently of the actions whose accurate sequential execution it controls. Thus, although the evolution of activity in the temporal context layer could well reflect associative chaining, temporal and characteristic effector order errors reflect both the precision of the mapping from the temporal context to the parallel planning layer and the process of selection during CQ. Thus, CQ models predict a strict factorization of sequential and item information, whereas RNN network models that are successfully trained to reproduce sequences do so by producing a mixed internal representation reflecting conjunctions of positions and items (Botvinick and Plaut, 2006).

Direct neurophysiological evidence in support of the CQ model has been obtained by Averbeck et al. (2002), who taught non-human primates to copy geometrical shapes presented on the computer screen while they recorded multi-unit activity from the prefrontal cortex, an area homologous to the inferior frontal cortex in the human brain (Bohland et al., 2010). Specifically, they characterized multi-unit patterns during the production of each segment in the shape-drawing sequence and then decoded the presence of each segment’s activity pattern during movement preparation and production. They found that the strength of each segment’s activity pattern at the end of the preparation phase corresponded to its respective position in the upcoming sequence (Figure 1D), as predicted by CQ models. However, it is not clear whether these activity patterns primarily reflected a graded preparation of specific motor elements in the sequence or a higher-level temporal or ordinal position signal (first, second, third, etc.) that might transfer across different motor elements.

Here we provide direct evidence for CQ in the human brain during the preparation of accurately timed finger sequences from memory using non-invasive whole-head recordings (magneto-encephalography [MEG]). Further, we demonstrate that the observed CQ pattern in the MEG signal during preparation primarily reflects the ordinal position of sequence items, largely independent of finger identity and fine temporal structure, and cannot be explained by a graded muscular pre-activation. Finally, we show that the fidelity of CQ correlates with behavioral accuracy across participants, suggesting that these neural representations are relevant for skilled sequence production and timing.

## Results

### Sequence Performance Shows Independent Temporal Transfer

**Figure 2:**
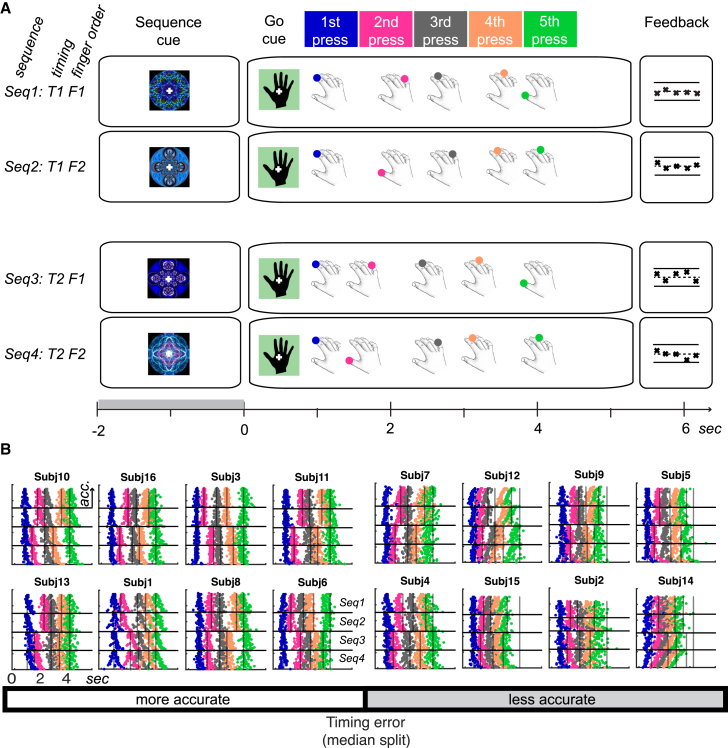
Task and Production of Sequences from Memory (A) Subjects were required to prepare and produce one of four finger press sequences, as indicated by a unique visual fractal cue, followed by a go cue. The four sequences were unique combinations of two finger orders (*F1* and *F2*) and two temporal or interval orders (*T1* and *T2*) and were generated randomly for each subject. Subjects received feedback on the finger and temporal accuracy of their five finger presses after each trial, with crosses (x) signifying correct and dashes (–) incorrect finger press responses across positions. The relative position of crosses on the y axis relative to a dashed midline indicates temporal accuracy. (B) Individual subjects’ raster plots show the timing of single button presses for each trial produced from memory after the go cue (*t* = 0) during the MEG session (target timing superimposed, black lines). The color code corresponds to the press position in (A). Trials for each sequence are grouped by sequence number and sorted by temporal accuracy, with the most accurate in the top to least accurate trials in the bottom rows. Participants are grouped into more accurate and less accurate based on a median split of their average temporal performance error and sorted within each group by accuracy (most accurate at the top left to the least accurate at the bottom right). Only trials with correct finger order were used for analysis.

Participants were trained for 2 days to associate four abstract visual cues with the production of four five-element finger sequences from memory following a “go” cue (Figure 2A). In the MEG session on the third day, the mean incidence of finger errors (trials with the wrong finger press order or incomplete sequences) across the whole group ranged from 0%–13% (mean, 2%; SD, 3%). Participants were median-split by the incidence of finger errors for later analysis, with more accurate participants producing, on average, 0.6% (SD, 0.3%) and the less accurate 3.7% (SD, 4%) trials with an incorrect finger order. Although, for the majority of participants, the production of the correct sequences was temporally aligned according to temporal interval orders *T1* and *T2* across finger orders *F1* and *F2*, respectively (Figures 2A and 2B), there was also a substantial variation of timing accuracy across participants. The mean temporal error from target interval structure (expressed as percent of target interval) across the whole group ranged from 10%–33% deviation from the target interval sequence (mean, 19%; SD, 7%; Figure 2B). Hence, participants were also grouped using a median split of interval deviation (temporal error) for later analysis, with more accurate participants showing, on average, 14% (SD, 3%) and the less accurate 25% (SD, 5%) absolute deviation from target intervals. On average, participants tended to produce finger press intervals 7% shorter than the respective target intervals, resulting in sequences that were slightly faster than the target sequence. Participants who were less accurate (larger absolute deviation from the target) were also more likely to produce shorter rather than longer intervals (*r* = −0.817, p < 0.001, two-tailed). Crucially, participants who made more finger errors also tended to be less accurate in their timing (*r* = 0.616, p < 0.011, two-tailed), suggesting that the differences in accuracy are driven by overall skill level rather than a strategic trade-off between temporal and finger accuracy.

**Figure 3:**
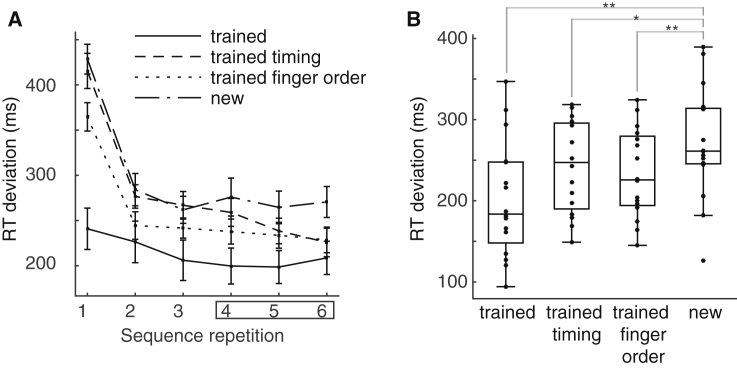
Independent Transfer of Timing and Finger Order to Untrained Finger Sequences (A) In a visually cued synchronization task, absolute RT deviation from finger cue was determined for each condition. Repeating sequences six times in the test phase yielded an immediate decrease of absolute RT deviation from target for trained sequences (green) relative to untrained sequences (black). Sequences with a trained finger order but an untrained timing (blue) also produced immediate benefits. Sequences with a trained timing but an untrained finger order (red) also showed behavioral advantages, but only after the first three sequence repetitions, suggesting faster behavioral acquisition of new sequences compared with a control condition (Kornysheva and Diedrichsen, 2014, Kornysheva et al., 2013). (B) Mean RT deviation across subjects after the first three sequence repetitions showed behavioral advantages for the trained sequence as well as the trained finger order and trained temporal transfer conditions compared with untrained sequences. ^∗∗^p < 0.01, ^∗^p < 0.05, one-sided t test. Error bars indicate SEM.

To evaluate sequence-specific learning, we conducted a post-training test on the day before the MEG session during which participants were asked to synchronize their respective presses to a visual finger cue, as in the first stages of training (STAR Methods). Participants showed significantly more accurate synchronization to visual sequences when they encountered trained sequences as well as sequences with a trained finger order or trained timing compared to untrained control sequences (Figures 3A and 3B). This result confirmed an independent representation of the temporal structure of sequences that can be utilized across finger sequences, in line with previous studies (Kornysheva and Diedrichsen, 2014, Kornysheva et al., 2013, Ullén and Bengtsson, 2003). Participants with more pronounced sequence-specific learning (quantified as the difference in synchronization accuracy to trained versus untrained sequences) produced larger inter-press intervals during the MEG session (*r* = 0.501, p < 0.001, two-tailed), suggesting that the tendency to compress the sequence was a marker of poor skill learning.

### MEG Evidence for Competitive Queuing

**Figure 4:**
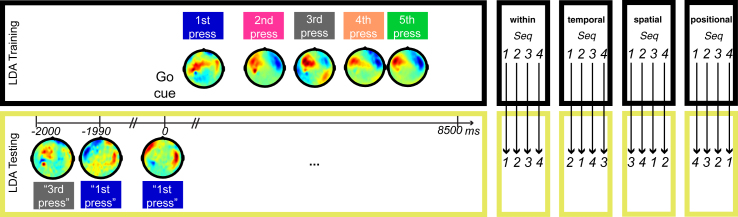
Linear Discriminant Analysis and Classification A Gaussian-linear classifier was trained to discriminate patterns of mean signal amplitude in a 10-ms time window preceding each of the five button presses across all sensors in a dataset consisting of correct trials only. Example mean signal amplitude values across the scalp are shown for a representative participant (temporal accuracy z = 0.45). The test dataset consisted of non-overlapping 10-ms time windows starting 2,000 ms before and ending 8,500 ms after the go cue (“within” the same sequences). For each time window in each trial, we calculated the probability of each button press. For illustration purposes, only the pattern with the highest probability is shown (inverted commas) for one example trial in the same participant. Further, we also trained and tested the classifier using sequences with the same timing but a different finger order (“temporal”), the same timing but a different finger order (“spatial”), and a different finger order and timing (“positional”), respectively. The same analysis was performed on the mean signal amplitude from the four concurrently recorded EMG channels (data not shown).

During the MEG scan, finger sequences were visually cued with an abstract shape for 1.8–2.2 s before a go cue. This provided participants with a short period to retrieve and prepare the corresponding sequence from memory prior to production. We used multivariate linear discriminant analysis (LDA) to characterize whole-head MEG activity patterns associated with the execution of each finger press. Specifically, we trained our classifier on the average MEG signal amplitude pattern across all sensors in a 10-ms window immediately preceding each finger press onset during sequence production and then applied the classifier to successive 10-ms time windows during sequence preparation to establish the posterior probability of each press-related pattern appearing during that period (Figure 4; see Figure S1 for complementary analyses using 5-, 20-, and 50-ms windows). We tested the main prediction of CQ models: that there is a stable and graded likelihood of decoding each item (i.e., finger press) during the preparation period, reflecting its position in the upcoming sequence. This stands in contrast to associative chaining or state-space models, which predict an increased likelihood of decoding the first item alone prior to sequence production. In addition, classification across different finger sequences (“temporal” and “positional” transfer; Figure 4) allowed us to distinguish the contribution of representations of specific motor acts in the parallel preparation layer from those reflecting a higher-order temporal context signal, whereas classification across different temporal sequences (“spatial” and “positional” transfer) allowed us to examine the extent to which the temporal context signal reflects positional or fine-temporal structure.

**Figure 5:**
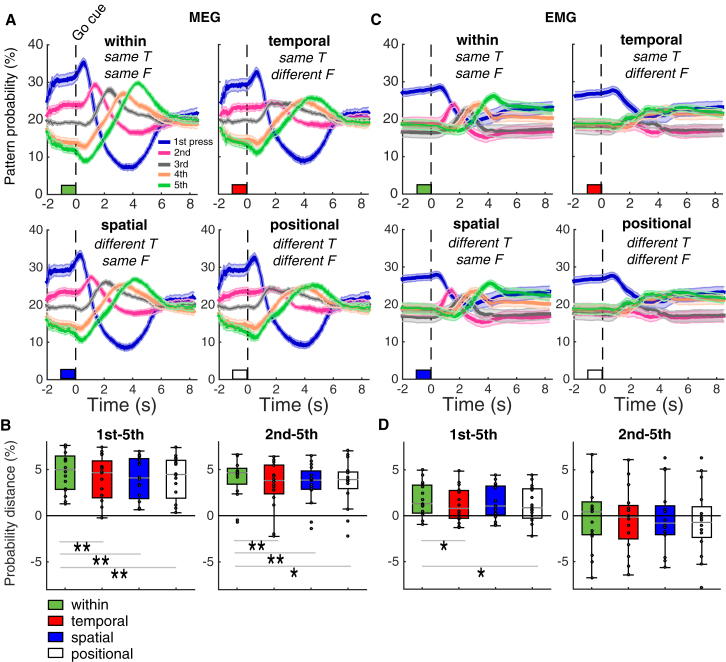
Neural versus Muscular Markers of CQ (A) MEG patterns. Trace plots display the probability of the first through fifth finger press patterns throughout preparation and production. Pattern probabilities “within” refers to training and testing within the same sequence trials. Press pattern probability traces were also calculated for training and testing across sequences with the same timing but a different finger order (“temporal”), the same finger order but a different timing (“spatial”), and a different finger order and timing (“positional”), respectively (Figure 3). (B) Boxplots show the average distance between consecutive press pattern probabilities in the final 1 s before the go cue across subjects considering first through fifth (left) and second through fifth press patterns (right), respectively. Note that, for each subject, the sequences always started with the same finger and target interval. Positive values indicate that the average relative strength of the press pattern probabilities tended to be in accordance with the temporal position in the sequence. Together, the MEG results suggest a CQ of sequential movement patterns during preparation that generalizes across sequences, but equally a significant reduction of CQ when classifying across sequences with different finger orders, timing, and both. (C and D) Trace plots display the probability of the 1st–5th EMG finger press patterns (C) and boxplots the average distance between consecutive EMG press pattern probabilities in the final 1 s before the “go” cue (D). In contrast to the MEG classification results, only the pattern for the first element in the sequence was elevated in muscular space, suggesting that, at the periphery, only the first element in the sequence was prepared before the go cue. ^∗∗^p < 0.001, ^∗^p < 0.05 (paired samples t tests, Bonferroni-corrected for three comparisons for first through fifth and second through fifth distances as well as MEG and EMG separately).

Our analysis demonstrated that, during sequence preparation, the probability of each finger press being decoded across time windows reflected its serial position in the upcoming sequence (Figure 5A, “within”), analogous to findings obtained using invasive electrophysiology recordings in macaques (Averbeck et al., 2002). Specifically, the mean pattern probability during the final 1 s of the preparation period was modulated by press position (*F*(1.71, 25.60) = 42.23, p < 0.001, *η*^*2*^ = 0.738; one-way repeated measures ANOVA, Greenhouse-Geisser-corrected, χ^2^ (9) = 37.56, p < 0.001). As predicted by the CQ hypothesis, pattern probabilities during this period were significantly higher for first versus second (*t*(15) = 4.62, p < 0.001), second versus third (*t*(15) = 4.57, p < 0.001), and third versus fourth (*t*(15) = 4.51, p < 0.001) finger presses, whereas the probability of decoding the fourth press was not significantly higher than that of the fifth (*t*(15) = 1.78, p = 0.19; one-tailed t tests according to the CQ hypothesis, Bonferroni-corrected for four comparisons).

### The Competitive Queuing Signal Primarily Reflects Serial Position

Next we aimed to establish whether CQ of press patterns during the preparation period was primarily driven by representations of effector identity (i.e., index finger, middle finger), versus effector-independent sequence timing (i.e., 550-ms interval, 650-ms interval), or ordinal position (i.e., first item, second item), which are indistinguishable when training and testing the classifier on data from a single learned sequence. To do so, we first examined whether CQ of neural signals during sequence preparation was preserved when classifying MEG patterns across sequences with the same target timing but a different finger order (temporal transfer). If neural CQ reflected the upcoming order of specific effectors (here, fingers) alone, then we would expect the accurate queuing of pattern probabilities to collapse because the upcoming finger order is rearranged relative to the training order in the second to fifth positions; i.e., the training pattern for a particular press position would reflect a different finger than that in the test pattern. In contrast, if the neural CQ signals during preparation included representation of sequence timing or ordinal position regardless of effectors, then CQ during the preparation phase should be upheld across sequences with a different finger order (i.e., exhibit temporal transfer).

This analysis revealed that CQ during the preparation period was qualitatively preserved across sequences with the same temporal intervals but differing finger order (Figure 5A, temporal). Specifically, mean pattern probability in the final 1 s of the preparation period was still modulated by press position (*F*(1.68, 25.15) = 26.97, p < 0.001, *η*^*2*^ = 0.643; one-way repeated measures ANOVA, Greenhouse-Geisser-corrected, χ^2^ (9) = 39.87, p < 0.001), with pattern probabilities at the end of the preparation period being significantly higher for first versus second (*t*(15) = 4.06, p = 0.002), second versus third (*t*(15) = 3.29, p < 0.001), and third versus fourth (*t*(15) = 3.97, p = 0.002) finger presses, whereas the probability of decoding the fourth press was again not significantly higher than that of the fifth (*t*(15) = 0.98, p = 0.68; one-tailed t tests according to the CQ hypothesis, Bonferroni-corrected for four comparisons). These results demonstrate that the CQ of sequence elements during preparation is only partially driven by finger identity but mainly derives from overlapping representations of sequential timing or ordinal position in a temporal context layer. They are also in line with significant advantages for learning sequences that retain the trained timing structure (Figure 3).

Next, we tested whether changing the fine timing structure between training and testing patterns while retaining the same ordinal finger sequence (spatial transfer) affected CQ. This analysis revealed that CQ during the preparation period was qualitatively preserved across sequences with the same finger order but differing temporal structure (Figure 5A, spatial). Specifically, the mean pattern probability during the final 1 s of the preparation period was still modulated by press position (*F*(1.61, 24.21) = 32.10, p < 0.001, *η*^*2*^ = 0.687, one-way repeated measures ANOVA, Greenhouse-Geisser-corrected, χ^2^ (9) = 45.11, p < 0.001), with pattern probabilities at the end of the preparation period being significantly higher for first versus second (*t*(15) = 4.22, p = 0.001), second versus third (*t*(15) = 3.57, p < 0.006), and third versus fourth (*t*(15) = 4.22, p = 0.001) finger presses, whereas the probability of decoding the fourth press was again not significantly higher than that of the fifth (*t*(15) = 1.69, p = 0.22; one-tailed t tests according to the CQ hypothesis, Bonferroni-corrected for four comparisons). These results are in line with significant advantages for learning sequences that retain the trained finger order structure (Figure 3).

In combination, these results showed that CQ of neural signals during the preparation period is preserved across finger orders and temporal structure, suggesting that it includes a high-level ordinal position code. To directly test this hypothesis, we investigated whether changing both the finger order and temporal structure between training and test patterns while retaining information about ordinal position in the sequence alone (positional transfer) would abolish CQ. Again, CQ during preparation was retained (*F*(1.62, 24.30) = 32.10, p < 0.001, *η*^*2*^ = 0.657, one-way repeated measures ANOVA, Greenhouse-Geisser-corrected, χ^2^ (9) = 41.19, p < 0.001), with mean pattern probabilities in the final 1 s of the preparation period being significantly higher for first versus second (*t*(15) = 4.05, p = 0.002), second versus third (*t*(15) = 3.56, p < 0.006), and third versus fourth (*t*(15) = 3.70, p = 0.004) finger presses, whereas the probability of decoding the fourth press was again not significantly higher than that of the fifth (*t*(15) = 1.58, p = 0.270; one-tailed t tests according to the CQ hypothesis, Bonferroni-corrected for four comparisons). These results confirm the manifestation of a finger- and timing-independent code for ordinal position during preparation that transfers across motor and temporal sequences.

Having established the preservation of CQ when classifying across sequences, we next sought to directly quantify any reductions related to the change of finger- or timing-related information. To this end, we directly compared the relative strength of neural CQ signals, quantified as the distance between consecutive press probabilities across the last 1 s of the preparation period in each trial (single trial median averaged across trials for each participant) for each of the classifiers (“within,” “temporal,” “spatial,” and “positional”). A repeated measures ANOVA with factors of “finger order” and “timing” changes revealed significant main effects of “finger order” (*F*(1.39, 15) = 12.15, p = 0.003, *η*^*2*^ = 0.447) and “timing” changes (*F*(1.67, 15) = 12.15, p = 0.002, *η*^*2*^ = 0.468) as well as a significant interaction between the two factors (*F*(1.92, 15) = 41.74, p < 0.001, *η*^*2*^ = 0.736). Direct comparisons between neural CQ during preparation for “within” sequence versus “temporal,” “spatial,” and “positional” transfer analyses, respectively, all yielded significant decreases of the probability distance during preparation (p < 0.0001, two-sample t test corrected for three comparisons). These findings demonstrate a significant attenuation of CQ when training and testing across sequences. They suggest that only the conjunction of temporal, spatial, and positional codes constitute a full-blown neural pattern for sequence preparation, consistent with the behavioral costs of changing the finger order and timing of a trained sequence (Figure 3).

Finally, to probe the transfer costs further, we trained the classifier on MEG data prior to presses with specific finger identities (in different ordinal positions and preceded by different temporal intervals), temporal intervals (in different ordinal positions and effected using different fingers), or ordinal positions (using different fingers and preceded by different temporal intervals) across all sequences. We then tested these classifiers on each sequence separately and averaged the results according to the position of the finger press or preceding temporal interval in that sequence to examine differences in CQ during the preparation period. Consistent with the findings described above, CQ was most pronounced when training on specific ordinal positions within each sequence, independently of finger identity or the preceding temporal interval (Figure S2).

In sum, these results indicate that the CQ during sequence preparation is primarily driven by a effector- and timing-independent code for ordinal position, presumably reflecting the overlapping successive representations in a temporal context layer.

### Decoding Dynamics during the Production Phase

In addition to the preparation phase, we also examined the dynamics of finger press probabilities during the production phase. Phasic execution-related peaks were markedly attenuated in analyses that classified across finger sequences (“temporal” and “positional” classifiers; i.e., when finger identity differed between training and test sequences) compared with the “within” and “spatial” sequence analysis (where finger identity was preserved between training and test sequences). Specifically, the mean decoding likelihood at the time of each finger press across the second to fifth presses was significantly higher in the “within” sequence analysis (production values cross-validated across trials; Figure S3) compared with “temporal” (*t*(15) = 4.71, p < 0.001, paired one-tailed t test) and positional (*t*(15) = 5.70, p < 0.001,) but preserved in “spatial” transfer analyses (*t*(15) = 0.82, p = 0.638, paired one-tailed t test, Bonferroni-corrected for three comparisons), which classified across sequences with the same finger order. However, it is important to note that the respective press probabilities were still significantly above chance (i.e., 20%) at the time of each respective finger press across all analyses (all p < 0.001, one-tailed t test against chance, Bonferroni-corrected for four comparisons). This suggests that, although finger identity had a stronger influence on decoding probabilities during sequence production than preparation, the finger-independent, position-related patterns observed during the preparation period were also utilized during sequence production.

### Competitive Queuing Is Not Driven by Muscle Activity

Although the neural signature of CQ described above suggests a top-down signal for the temporal planning of finger sequences, it is still possible that this pattern is partly driven by a weighted activation of muscles at the periphery so that the muscle synergy activations related to each finger movement are weighted before production according to their occurrence. Hence, using the same LDA procedure as for the MEG data training and classification (Figure 4), we examined data obtained from muscles of the right hand (*flexor carpi radialis*, *abductor polices brevis*, *abductor digiti minimi*, first dorsal *interossei*) concurrently with the MEG recording. This analysis revealed that, during preparation, only the pattern probability for the first finger press in the sequence, performed by the same finger across all four sequences, was elevated above those for the other sequence elements.

Specifically, the mean electromyography (EMG) pattern probability during the final 1 s of the preparation period was modulated by press position for the “within” sequence analysis (i.e., training and testing the classifier on MEG data from the same pattern; *F*(4, 60) = 7.44, p < 0.001, *η*^*2*^ = 0.332; one-way repeated measures ANOVA) as well as “temporal” (*F*(2.74,41.17) = 5.27, p = 0.005, *η*^*2*^ = 0.260; one-way repeated measures ANOVA), “spatial” (*F*(4, 60) = 7.39, p < 0.001, *η*^*2*^ = 0.330; one-way repeated measures ANOVA), and “positional” (*F*(4, 60) = 5.88, p < 0.001, *η*^*2*^ = 0.282; one-way repeated measures ANOVA) transfer analyses. However, in contrast to the MEG data, this effect was driven purely by the elevation of the first press pattern probability in the sequence, which was the same across all sequences within subjects (first versus second “within”: *t*(15) = 3.882, p = 0.003; “temporal”: *t*(15) = 3.583, p = 0.005; “spatial”: *t*(15) = 4.106, p = 0.003; “positional”: *t*(15) = 3.886, p = 0.003), with no other differences between adjacent press probabilities reaching significance in any analysis (p > 0.775, one-tailed t tests according to the CQ hypothesis, Bonferroni-corrected for four comparisons). Finally, the distance between consecutive press probabilities in the MEG data did not correlate with the distance in the EMG data in either the “within” (*r* = 0.246, p = 0.179), “temporal” (*r* = 0.321, p = 0.112), “spatial” (*r* = 0.182, p = 0.250), or “positional” (*r* = 0.128, p = 0.319) classification analyses. Although the data provide strong evidence for muscular preparation of the first press before the go cue, we could find no evidence for weighted muscular synergies driving the CQ pattern in the CNS during sequence preparation.

### Competitive Queuing Is Not an Artifact of Temporal Proximity

It is conceivable that the pattern probability gradient observed here during the preparation period arises simply from auto-correlation in the MEG time series and the temporal proximity of training and testing time windows rather than the CQ of sequence-related patterns. Specifically, this alternative hypothesis assumes that there is a slow-moving brain state across both preparation and production phases within each trial, resulting in the MEG signal being more similar during time windows that are close together (i.e., preparation period and first press) than those further apart (i.e., preparation period and fifth press). If true, then there would be a gradient of pattern probabilities during movement preparation, reflecting the relative temporal offset between the preparation period (on which the classifier was tested) and the time of each finger press (on which the classifier was trained).

To examine this possibility, we conducted several control analyses. First, we examined decoded press probabilities around the time that the sequence cue appeared, indicating that sequence preparation should begin 1.8–2.2 s before the go cue. If these decoded press probabilities simply reflected temporal proximity to the corresponding press, then the appearance of this cue should not change the press probabilities. However, we found an abrupt increase in the differences between successive press probabilities at the onset of the visual sequence cue (Figures S4A and S4B), with CQ becoming more pronounced as full information regarding the order and timing of finger presses becomes available.

Next, we examined the slope of each finger press probability time series during the preparation period. If they simply reflect temporal proximity to the upcoming press, then they should each exhibit a constant positive slope over the preparation period. Conversely, if the press probabilities reflect the stable CQ of elements in the upcoming sequence, then they would not be expected to change over the preparation period. Consistent with the latter hypothesis, none of the press probabilities for the second to fifth items in the upcoming sequence showed a significant positive slope during the final 1 s of the preparation period (all p > 0.99, one-sample tests against zero, Bonferroni-corrected for 5 comparisons; Figure S4C), although the probability slope for the first finger did approach significance (p = 0.07).

Finally, we examined correlations between the MEG activity patterns on which each finger press classifier was trained and those during every other time bin across the preparation and production periods (Figure S5A). If press probabilities during sequence preparation arose as a result of slow drift in the MEG signal, then correlations between the ongoing MEG activity and the training patterns should be positive and increase gradually with temporal proximity to the relevant finger press. Conversely, we found that activity patterns for the second to fifth finger presses were anti-correlated with MEG activity patterns during the preparation period, with correlation coefficients only becoming positive after the onset of sequence production (see Figure S5B for the EMG pattern similarity analyses and Figure S5C for MEG and EMG production pattern similarity matrices used to train the classifier). Overall, these findings are consistent with stable differences in press probabilities arising as a result of CQ shortly after the sequence cue appears, rather than a slow increase in press probabilities arising as a result of increasing temporal proximity to the respective classifier training windows.

### Competitive Queuing Predicts Behavioral Accuracy

Next, we asked whether the degree of neural CQ during sequence preparation was relevant for the subsequent production of the finger sequences retrieved from memory. Specifically, we examined whether the average distance between successive pattern probabilities during the final 1 s of the preparation period (immediately preceding the go cue) predicted the subsequent finger order and timing accuracy across trials in the MEG session (STAR Methods).

**Figure 6:**
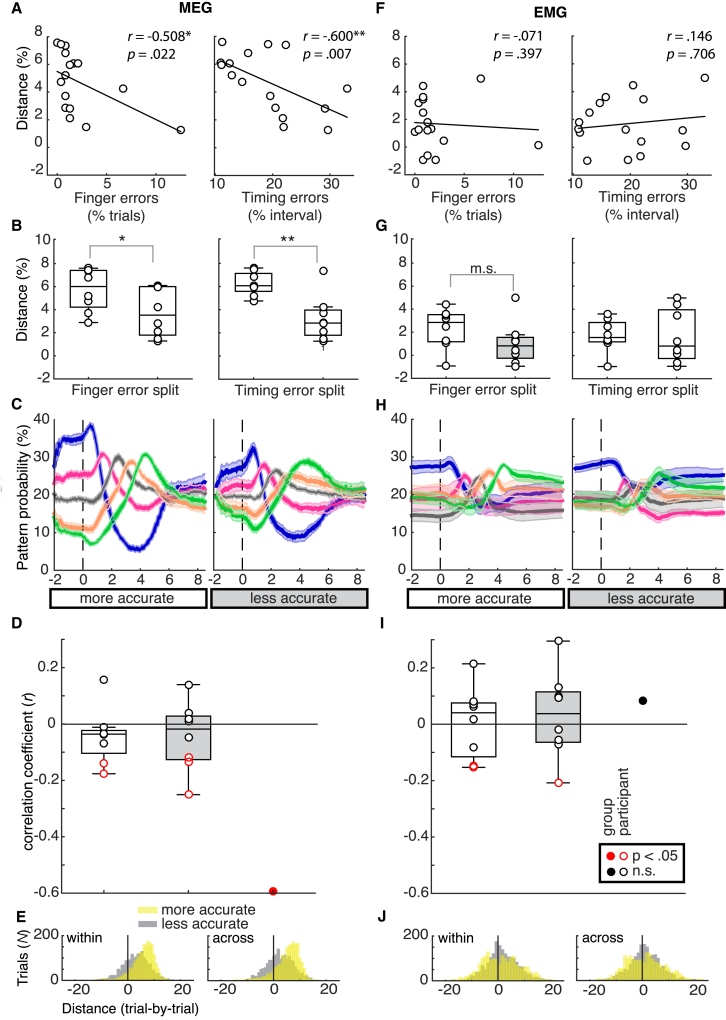
Correlation with Behavior (A) Overall, both the incidence of trials with finger press errors (left) and the mean temporal error from target intervals (in percent, right) during the MEG session correlated negatively with the distance between consecutive MEG pattern probabilities. The distance was extracted from the end of the preparation period in the “within” sequence classification (first through fifth) as a proxy of the strength of CQ. (B) The median split based on finger press and timing errors revealed significantly stronger CQ for subjects with lower finger and timing error (higher spatial and temporal accuracy). (C) Trace plots as in Figure 5A, grouped according to the median split by timing error (see Figure S6 for grouping by percentage of trials with finger press errors). (D) Trial-by-trial pattern distance probabilities and temporal performance were correlated in only five of 16 subjects. This finding suggests that the strength of neural CQ during sequence planning predicted overall but not trial-to-trial performance in the subsequent execution period. Participants' correlation coefficients are grouped according to a median split by timing error, white shading signifying more and gray shading less accurate participants. (E) Histograms show distributions of trial-by-trial pattern queuing distances during preparation, collapsed across more accurate and less accurate participants, respectively. Positive values reflect that most trials showed the relative strength of the press pattern probabilities to be in accordance with the temporal position in the sequence trial by trial, in particularly for more accurate subjects. (F–H) Association between pattern probability spread and behavior did not hold up for preparatory EMG patterns. Specifically, (F) the pattern probability spread did not correlate with finger press errors (left) or temporal error, (G) showed no significant differences in a median split based on finger press or temporal errors, or (H) corresponding trace plots as in Figure 5A, grouped according to the median split by temporal error. (I and J) No association of EMG preparatory patterns and behavior was found in (I) trial-by-trial pattern distance probabilities and temporal performance and (J) trial-by-trial pattern distances during preparation, when collapsing across more accurate and less accurate participants. ^∗∗^p < 0.01, ^∗^p < 0.05, *m.s.* p *=* 0.09, one-sided t test and linear correlations in line with the directional hypothesis for behavior and CQ patterns. Two-sided t tests and correlations were also significant at p < 0.05. Error bars indicate SEM.

Consistent with the predictions of CQ models, our findings suggest that participants with a larger mean distance between adjacent press probabilities according to their sequence positions tended to make fewer finger errors during the MEG session (*r* = −0.508, p = 0.022; median split by finger accuracy: *t*(15) = 1.99, p = 0.033) and produce smaller temporal errors relative to the target timing structure of sequences (*r* = −0.600, p = 0.007; median split by temporal accuracy: *t*(15) = 3.87, p < 0.001; Figures 6A and 6B). In particular, the probability dynamics shown in Figure 6C illustrate striking differences in the fidelity of CQ during sequence preparation between participants with more and less accurate behavioral performance (median split by timing accuracy; see Figure S6 for median split by finger order accuracy) as well as the relative preservation of phasic response curves during the serial execution period. Crucially, this correlation with behavioral accuracy was also unique to MEG patterns. Despite the elevation of the first EMG press pattern probability prior to the go cue, which could have played a role in subsequent sequence production, the fidelity of CQ in EMG patterns did not show any significant relationship with overall points gained or the size of temporal errors (Figures 6F–6H).

In contrast to the correlation between temporal accuracy and average pattern probability distance across participants, we did not find evidence that pattern probability distance during preparation predicted temporal accuracy during subsequent production on a trial-by-trial basis. Within participants, the trial-by-trial correlation coefficients ranged from *r* = −0.251 to *r* = 0.157 (SD = 0.107), with a predicted negative correlation being significant at p < 0.05 in only 5 of 16 participants (Figure 6D, grouped by timing accuracy). Accordingly, participants with a more pronounced neural CQ pattern during sequence preparation had better overall performance in the sequence production phase. However, despite the presence of CQ at the trial-by-trial level (Figure 6E), this neural signal did not guarantee high execution accuracy on each trial, which may be influenced by other downstream processes for motor implementation.

For the EMG patterns, in line with the group analysis, we did not find any consistent significant trial-by-trial correlations between the EMG pattern probability distance and temporal accuracy. Within participants, the trial-by-trial correlation coefficients ranged from *r* = −0.208 to *r* = 0.295 (SD = 0.137), with a predicted negative correlation being significant at p < 0.05 in only 3 of 16 participants (Figure 6I, grouped by timing accuracy) with, on average, no CQ at the single-trial level (Figure 6J). Therefore, in the case of EMG data, there was no evidence of the median probability distance associated with performance, neither across nor within participants (trial-by-trial).

### Competitive Queuing Originates from Parahippocampal and Cerebellar Sources

**Figure 7:**
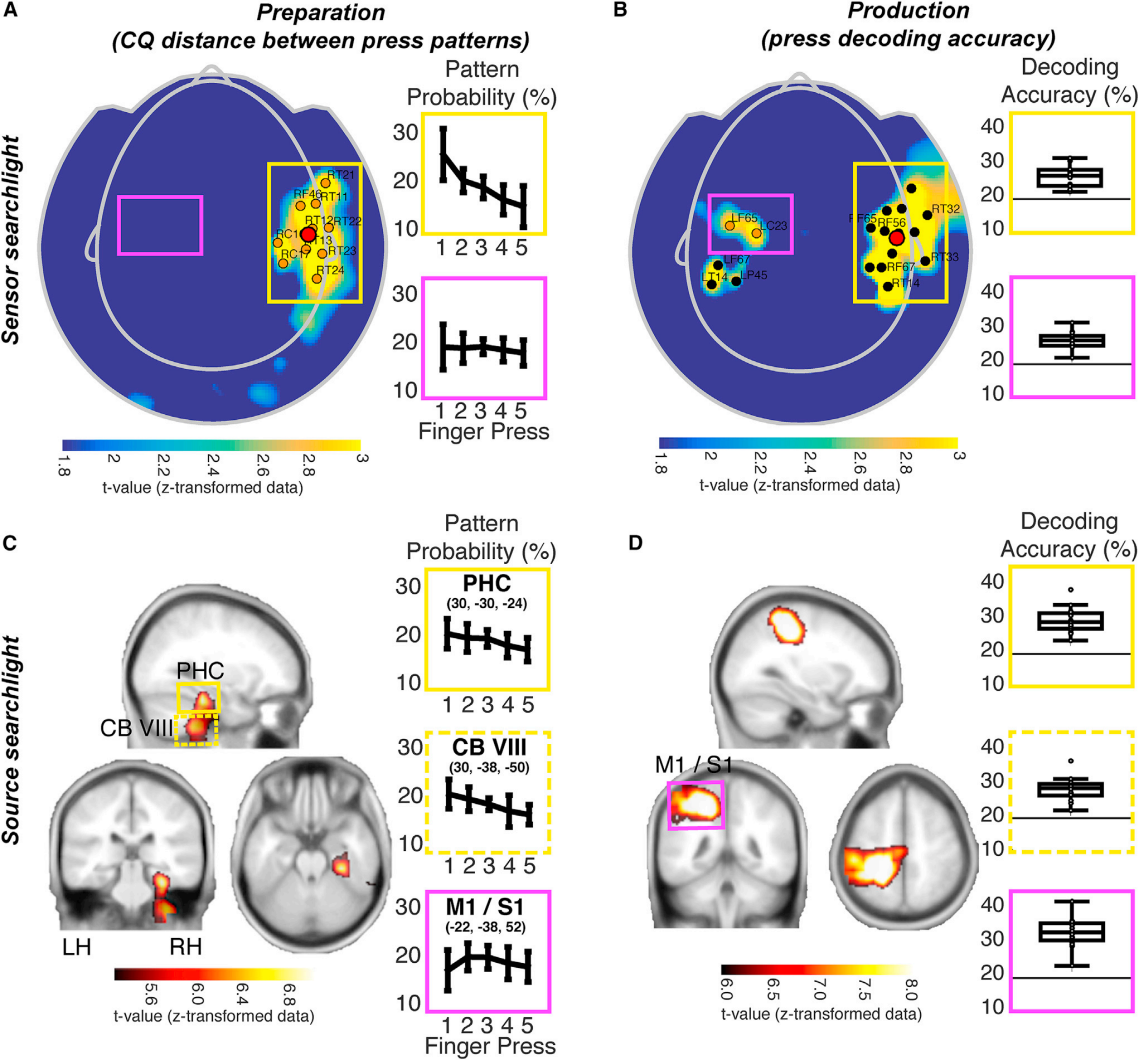
Significant Sensors and Sources with the Most Pronounced CQ Strength during Preparation (A) CQ distance during preparation (sensor level). A sensor-level searchlight analysis demonstrated that CQ during preparation was most pronounced in the right temporal sensors (p < 0.01, uncorrected). The red dot shows the centroid for the right cluster (masked by right frontal [RF], right central [RC], and right temporal [RT] sensors). (B) Decoding accuracy of finger presses during production (sensor level). Searchlight analysis revealed that sensors driving the differences between the press patterns during production were located above the left (contralateral) sensorimotor sensors in addition to right temporal sensors. The red dot shows the centroid for the right cluster (masked by RF, RC, and RT sensors). (C) CQ distance during preparation (source level). The within-sequence CQ distance searchlight analysis at the source level revealed the right parahippocampal area and right (ipsilateral) cerebellar lobules V and VIII as likely sources of CQ during preparation. Data are plotted as a t-statistic at a threshold of *t*(15) > 5.24, p < 0.05 (FWE-corrected at the voxel level) centered at the peak voxel (MNI coordinates: 30, −30, −24). Line graphs show corresponding pattern probabilities during preparation from peak voxels in significant clusters identified in the respective analyses presented in (C) and (D), specifically the right parahippocampus (yellow), right cerebellar lobule VIII (yellow dashed), and left sensorimotor neocortex (magenta). (D) Decoding accuracy of finger presses during production (source level). Searchlight analysis revealed that the differences between the press patterns during production were driven by the left (contralateral) sensorimotor neocortex. Data are plotted as a t-statistic at a threshold of *t*(15) > 5.93, p < 0.05 (FWE-corrected at the voxel level) centered at the peak voxel (MNI coordinates: −22, −36, 52). Boxplots show individual decoding accuracies in peak voxels of interest as in (C), suggesting that, despite not being significant at the whole-brain level, temporal sensors remained relevant during sequence production, with above chance (20%) decoding accuracies. Error bars indicate SEM. V, lobule V; VIII, lobule VIII; CB, cerebellum; LF, left frontal; LH, left hemisphere; LP, left parietal; LT, left temporal; M1, primary motor cortex; PHC, parahippocampal area; RC, right central; RH, right hemisphere; RT, right temporal; S1, primary sensory cortex.

Finally, we sought to identify the neural origins of CQ during preparation in MEG sensor and source space. To this end, we used a searchlight analysis to determine where the CQ signal during the preparation period was strongest. Specifically, we quantified the average distance between consecutive press probabilities at a single time point 0.5 s before the go cue in each trial, after training and testing our classifiers on data from the same finger press sequences (“within” sequence analysis). These median distance values were then z-transformed across all sensor and voxel searchlights within each participant prior to group-level statistical analysis. The most pronounced effect of CQ during preparation appeared in the right temporal sensors (*t*(15) = 3.58, p < 0.01, uncorrected; Figure 7A). In line with this finding, the same analysis conducted in source space showed a significant CQ distance effect to originate from a single cluster (p_*cluster*_ < 0.001) comprising the right temporal cortex, specifically the parahippocampus, extending ventrally into the fusiform area (Montreal Neurological Institute [MNI] coordinates of peak voxel: 30, −30, −24, *t(15)* = 6.94, p = 0.005, p value family-wise error [FWE]-corrected at voxel level) and the right (ipsilateral) cerebellum, specifically lobules VIII (MNI coordinates of peak voxel: 34, −30, −50, *t(15)* = 6.51, p = 0.009) and V (MNI coordinates of peak voxel: 64, −46, −30, *t(15)* = 5.72, p = 0.046; Figure 7C).

In addition, to localize the sources of the press-related training patterns (that is, the representations of execution of a specific finger press within a given sequence), we trained and tested our classifier on MEG data obtained 10 ms prior to each finger press within each motor sequence using a 5-fold cross-validation procedure. As before, decoding accuracy values were z-transformed across sensors and voxel searchlights within each participant prior to group-level statistical analysis. Consistent with prior findings (Wiestler and Diedrichsen, 2013, Wiestler et al., 2011), the accuracy of finger press decoding was most pronounced in the sensors located above the left sensory and motor areas contralateral to the moving hand and the right temporal sensors (*t*(15) = 3.11, p < 0.01, uncorrected; Figure 7B), the latter partly containing the same significant sensors as in the CQ analysis from the preparation period. At the source level, we found a large significant cluster (p_*cluster*_ < 0.001) comprising contralateral primary sensory and motor regions with peaks in the primary sensory cortex (MNI coordinates of peak voxels: −22, −38, 52, *t(15)* = 11.19, p < 0.001 and −54, −30, 40, *t(15)* = 8.87, p = 0.001), extending medially into the cingulate cortex (MNI coordinates of peak voxel: −14, −28, 44, *t(15)* = 8.96, p = 0.001; Figure 7D).

Finally, to gain a more detailed picture of the complementary contributions of these regions during sequence preparation and production (Figures 7A–7D, line and boxplots), we examined CQ during the preparation phase and finger press decoding accuracy from the production phase in each group of significant sensors and peak voxels of significant clusters reported above. Interestingly, the finger press decoding accuracy in the right temporal sensors (*t*(15) = 8.66, p < 0.001) as well as the right parahippocampal (*t*(15) = 10.73, p < 0.001) and cerebellar sources (*t*(15) = 10.73; p < 0.001, one-sided t tests against chance level) identified by the CQ analysis during the preparation phase showed above-chance finger press decoding accuracy in all sixteen subjects during the production phase. This suggests that activity patterns in parahippocampal and cerebellar regions during preparation also contained information about the finger press being executed during sequence production, although the size of the effect was eclipsed by the concurrent representation of finger press identity in contralateral S1 and M1 regions. In contrast, the probabilities in central sensors as well as contralateral S1 and M1 did not show any evidence of a CQ gradient during preparation (sensor level: one-way repeated measures ANOVA, *F*(2.32, 17.77) = 0.37, p = 0.72, *η*^*2*^ = 0.024; Greenhouse-Geisser-corrected, χ^2^ (9) = 21.58, p = 0.01; source level: *F*(3, 32.18) = 1.82, p = 0.16, *η*^*2*^ = 0.108; Greenhouse-Geisser-corrected, χ^2^ (9) = 18.71, p = 0.03), suggesting that central and contralateral neocortical sensorimotor areas did not contribute to establishing the temporal order of finger presses before execution. In sum, our results indicate a special role of parahippocampal and effector-related cerebellar sources in establishing a CQ gradient during sequence preparation and its utilization during sequence execution, whereas contralateral primary sensorimotor sources appear to contribute to the task during sequence execution only.

## Discussion

Using non-invasive neurophysiological recordings (MEG) in combination with multivariate pattern classification (LDA), we provide direct evidence for parallel CQ of planned sequential actions in humans. Our study extends previous findings in animal (Averbeck et al., 2002) and computational models (Botvinick and Plaut, 2006, Burgess and Hitch, 2005, Hartley and Houghton, 1996, Henson, 1998, Rhodes et al., 2004) in several directions. We show that neural CQ signals reflect an abstract template for ordinal position that is transferable across movement sequences and that the strength of CQ during preparation predicts the participant’s skill level during production. In addition, we localize the neural CQ signal during preparation to ipsilateral (right) parahippocampal and cerebellar regions and the production signal to the contralateral sensorimotor neocortex. These findings imply that sequential learning is factorized into representations of ordinal structure and specific movements, which are combined within a CQ mechanism.

### Neural Competitive Queuing in Humans

Despite differences in methods and species, our results bear remarkable resemblance to electrophysiology data obtained from macaques (Averbeck et al., 2002). Specifically, our data suggest that the elements constituting a movement sequence are prepared in parallel, with the corresponding pattern probabilities being weighted by their respective position in the subsequent sequence. After the go cue, these pattern probabilities transitioned into phasic increases reflecting the serial execution of the finger presses.

Our data are at odds with an alternative model of motor sequence control that has dominated the field in the last decade, implying that skilled sequences such as handwriting or tapping sequences (e.g., Morse code production) are controlled by serial state-space trajectories in RNNs mapped onto motor actuators downstream (Goudar and Buonomano, 2018, Laje and Buonomano, 2013). According to these models, we would not expect to find the respective population states for each movement to be reinstated before sequence initiation. An exception is the superpositional coding of sequence elements at the onset of sequence recall produced using an RNN model by Botvinick and Plaut (2006). Their model was able to produce population activity patterns relating to each position in the sequence and exhibit a summation of these patterns at the onset of the sequence without an explicit CQ architecture. This renders the RNN encoding effectively “parallel” and, thus, compatible with both the probability gradient for sequence elements during preparation shown by Averbeck et al. (2002) and our findings (Figure 5A).

It is possible that both serial chaining and parallel queuing of sequence representations co-exist in the nervous system. Accordingly, their utilization may be determined by the kinematic features of the motor sequence. Specifically, in contrast to the current discrete motor sequence (involving temporal gaps in between movements), continuous and overlapping sequences are prone to be encoded as integrated synergies, with movement timing emerging from state-dependent control; e.g., position- or velocity-dependent information (Conditt and Mussa-Ivaldi, 1999, Diedrichsen et al., 2007, Ivry et al., 2002, Zelaznik et al., 2005). Notably, most skilled actions in everyday life, such as speech, handwriting, and tool use, involve a wide range of discrete and continuous movements, necessitating the combination of these two control modes. Here future studies need to establish how different sequence types employed in these everyday domains are segmented and prepared at the neural level.

Finally, these theoretical accounts can be integrated in principle. Multiunit recordings during the preparation and production of reaching movements in non-human primates have revealed that changes in neural state related to planning occur in the null sub-space; i.e., a neural state that is distinct from the state during production, consistent with RNN predictions (Kaufman et al., 2014). Accordingly, Remington et al. (2018) proposed that such null-subspaces for each movement may be utilized to cue each element in the sequence independently. This suggestion, in principle, allows for a parallel control of discrete action sequences within the RNN framework.

### Competitive Queuing Reflects Finger-Independent Planning of Ordinal Position

We assessed whether the CQ pattern during preparation was driven by the preparation of specific movements in the sequence or by an action-independent template for the upcoming sequence, as predicted by models of serial recall within the framework of CQ (Bullock, 2004, Burgess and Hitch, 1999, Burgess and Hitch, 2006, Hartley and Houghton, 1996). By classifying within and across sequences with different finger orders and timing (Figure 4), we showed that the CQ signal during preparation was largely preserved under these circumstances, whereas the phasic increases in finger press probability during sequence production were distorted after the effectors diverged (second to fifth presses). This indicates that the training patterns, corresponding to mean activity in a 10-ms period before each registered finger press, contained information on the ordinal position in the sequence in addition to the effector information and that this information was retrieved from memory in the sequence planning stage.

The retrieval of an abstract template for ordinal position during movement planning is in line with models suggesting that the temporal evolution of the sequence is established by input from a reproducible temporal context signal to a parallel planning layer (Burgess and Hitch, 2005, Burgess and Hitch, 1999, Hartley and Houghton, 1996, Hartley et al., 2016). Furthermore, independent behavioral transfer of trained sequence timing to new finger orders in current (Figure 3) and previous studies in humans (typing, vocal sequences) and animal models (birdsong) (Ali et al., 2013, Bengtsson et al., 2005, Binder et al., 2014, Kornysheva and Diedrichsen, 2014, Kornysheva et al., 2013) supports the notion that temporal sequence features were encoded separately from the specific movements. This cannot be easily reconciled with classical associative chaining models or the idea of a single integrated spatio-temporal neural trajectory where the serial position is inextricably linked to the item (here, finger), such as in RNN models in which timing is an emergent property of the population trajectory. However, RNN models are, in principle, well-suited to serve as neural clocks (Buonomano and Laje, 2011) and have been shown to acquire superpositional coding of the list elements activated at the onset of recall (Botvinick and Plaut, 2006). Thus, the reported CQ of sequence element probabilities before movement production could primarily reflect a gradient of superimposed patterns encoding each temporal position in the sequence. Effectively, this mechanism implies the reinstatement of a high-level sequential plan during sequence preparation that can be utilized across different movement sequences in a modular manner.

We found that the most pronounced pattern of CQ during preparation originated in the right parahippocampal area. Neuroimaging studies have shown the recruitment of hippocampal and parahippocampal regions during motor sequence learning tasks, specifically those involving sequences of discretely timed movements, including those learned implicitly (Lungu et al., 2014, Schendan et al., 2003, Steele and Penhune, 2010). Moreover, parahippocampal activity has been reported to correlate specifically with the accuracy of temporal tapping patterns involving a sequence of short and long intervals (Steele and Penhune, 2010). Our results substantiate the broader involvement of hippocampal and parahippocampal areas in the timing of sequences (Jacobs et al., 2013, Kraus et al., 2013), and more specifically in the temporal succession or ordinal structure of sequences (Friston and Buzsáki, 2016, Manns et al., 2007). The temporal context signal itself could reflect the output of hippocampal “time cells” (Kraus et al., 2013). The observed graded press probabilities according to ordinal position in the sequence suggest that the first several press representations are all simultaneously active, with different levels of activity, as driven by connections from the overlapping representations in the temporal context layer corresponding to specific ordinal positions (Figures 1A–1C). Importantly, these can be shared across different sets of movement sequences (that is, across mappings to the items in the parallel planning layer established through Hebbian learning), enabling a higher-level sequential template (Burgess and Hitch, 1999, Burgess and Hitch, 2006).

Further, source reconstruction indicated the involvement of ipsilateral cerebellar lobules V and VIII, which have been shown to hold sensory and motor representations of fingers of the ipsilateral hand in humans (Wiestler et al., 2011). This suggests that the cerebellum works in concert with parahippocampal areas to achieve the queuing of actions during sequence preparation. Specifically, we speculate that, while the parahippocampal areas may retrieve a more abstract sequential plan for the sequence (Friston and Buzsáki, 2016), the representation of the actions themselves may be set by effector-specific cerebellar circuits in the form of a fine-grained spatio-temporal forward model of the finger sequence before onset of the production phase (Gao et al., 2012, De Zeeuw et al., 2011). Notably, activity attributed to the parahippocampal area and the cerebellum showed above-chance accuracy in decoding the sequence presses during the production period, whereas the reverse, neocortical sensorimotor regions showing CQ of finger press patterns during preparation, did not hold true. This dissociation suggests that the structure of the upcoming sequence is pre-specified outside of the neocortical regions that generate the movements and continues to be utilized during production (corresponding to temporal context and parallel planning layers in Figure 1A), whereas the regions representing the movement synergies are involved during production only (items in the competitive choice layer in Figure 1A).

Despite the presence of a high-level template for ordinal position across sequences, we found the strength of the queuing pattern during preparation to be diminished significantly when classifying across sequences with a different finger order or timing or both. These results suggest that representations that integrate ordinal position with specific movements and precise timing are also present, even at the stage of preparation.

Taken together, our data suggest that the brain learns the task structure and factorizes behavior into specific actions, their temporal structure, and ordinal position. Associating actions with a sequential position and selecting among them by CQ may be sufficient to generate skilled sequence production. This factorization makes learning more efficient and allows for transfer of a learned structure to new motor sequences.

### Strength of Competitive Queuing during Preparation Predicts Performance

Participants who achieved a higher skill level in the sequence production from memory showed a larger separation between adjacent press-related pattern probabilities during sequence preparation; i.e., the CQ pattern was more prominent in participants who made fewer finger and smaller timing errors (Figures 6A–6C). This is in line with the architecture of CQ models, in which more pronounced differences in the parallel planning layer (Figure 1) reduce the chance of pressing a finger at the wrong time during sequence production.

Remarkably, the significant correlation between overall performance and mean CQ strength contrasted sharply with the absence of a trial-by-trial correlation within participants. This dissociation is a possible indication that the strength of CQ during sequence preparation is a neural strategy adopted by, on average, more skilled performers. Although this neural strategy may be beneficial, it does not guarantee accurate performance on each trial, due to downstream modulations occurring between planning and motor implementation. Nonetheless, CQ was present on a trial-by-trial basis, which excludes the possibility that CQ is an artifact of averaging across trials with single elevated press probabilities. Specifically, participants who had reached a better behavioral performance tended to have more trials showing clear CQ than less skilled participants (Figure 6D).

The current non-invasive measure of CQ during sequence planning in humans as well as the link between overall CQ strength and performance provides a promising step toward the identification of markers for skilled sequence preparation. Determining the amount of separation between sequence elements before movement production may be useful in the context of neurofeedback training to improve performance in patients with higher-order motor impairments affecting sequence initiation and fluency, e.g., stuttering, dyspraxia, and occupational dystonia. Finally, our approach has the potential to advance the development of brain-machine interfaces for paralyzed patients. Previous studies have looked at predicting single targeted movements (Hochberg et al., 2006) or sequences up to two movements at a time; e.g., from invasive recordings (Shanechi et al., 2012). The current non-invasive method may assist the readout of a multi-element sequence during the planning period with the aim of achieving more fluent control of external devices for skilled sequence production, such as a virtual keyboard or tool use via an intelligent prosthesis.

## STAR★Methods

### Key Resources Table

**Table:** 

REAGENT or RESOURCE	SOURCE	IDENTIFIER
**Deposited Data**		

Mendeley Data	This paper	https://doi.org/10.17632/fxbmm66cr6.1

**Software and Algorithms**		

MATLAB 2016b	MathWorks	https://www.mathworks.com
Cogent	Cogent Developers	http://www.vislab.ucl.ac.uk/cogent.php
SPM12 FIL	FIL Methods Group	https://www.fil.ion.ucl.ac.uk/spm
Fieldtrip	Donders Institute for Brain Cognition and Behavior	http://www.fieldtriptoolbox.org/
LDA	Kornysheva and Diedrichsen, 2014	https://doi.org/10.7554/eLife.03043.018

### Contact for Reagent and Resource Sharing

Further information and requests for resources should be directed to and will be fulfilled by the lead contact, Dr. Katja Kornysheva (e.kornysheva@bangor.ac.uk).

### Experimental Model and Subject Details

#### Participants

Sixteen right-handed healthy adults (9 females; mean age 24.4 years, SD: 4.9) with normal or corrected-to-normal vision participated in this experiment which included two days of training and one MEG session. Five additional participants participated in the study but had to be excluded as follows: two participants due to poor performance at the end of the training session or during the MEG session (error rate > 40%), one participant due to the absence of sequence-specific learning at the end of training, one participant due to technical issues with the MEG system and one due to discomfort during the MEG session leading to the termination of the experiment. All participants gave written informed consent to participate. The study was approved by the University College London Research Ethics Committee for Human-Based Research (UCL Ethics ID: 1338/006, Data Protection: Z6364106/2011/10/25). All participants were financially compensated for their participation.

### Method Details

#### Stimuli and behavioral task

Stimuli were presented via a digital LCD projector (brightness = 1500 lumens, resolution = 1024 × 768 pixels, refresh rate = 60 Hz) onto a screen (height = 32 cm, width = 42 cm, distance from participant = 70 cm) that was parallel to the participant’s face inside a magnetically shielded room using the Cogent (http://www.vislab.ucl.ac.uk/cogent.php) toolbox running in MATLAB (The MathWorks, Inc., Natick, MA). Participants had a similar setup during behavioral training involving the same response device (5 buttons, Current Designs), with the visual stimuli shown on the computer screen directly and the participants being seated at an office desk. Stimuli were symmetrical abstract visual fractals. For each participant the four sequence cues were randomly selected and assigned to the sequences from a pool of sixteen fractals. Each trained sequence consisted of five-finger presses (finger order) and five target intervals (550, 650, 800, 983, 1300ms) between finger presses (timing or temporal interval order), respectively. Sequence construction followed a factorial design, with two orders of temporal intervals (*T1 and T2*) and two finger order sequences (*F1 and F2*) resulting in four unique temporally structured finger sequences (Figure 2A), generated randomly for each participant. Importantly, however, the finger identity and target timing for the first press in each sequence was preserved across all four sequences learned by each participant.

Participants were presented with a feedback screen after each trial showing the number of cumulative points across the whole experiment, as well as feedback on whether they pressed the correct finger at the correct time. Participants received two points per trial for a correct finger sequence with a temporal deviation from target timing of less than 30%, one point for a correct finger sequence with a temporal deviation of less than 60% and zero points in any other case. During the first two training days participants were presented with an auditory sound concurrently with the feedback screen, which indicated 0-2 points. No auditory feedback was presented during the MEG session on day 3.

Training on day 1 consisted of 7 instructed blocks containing only trials in which the sequence was cued by a circle appearing on the target finger at the target timing after the ‘Go’ cue to which the participants had to synchronize (168 trials), 7 mixed blocks (56 instructed and 112 from memory trials following each other in a blocked 1:2 pattern, respectively) and 7 blocks with sequences produced from memory (168 trials). Training on day 2 consisted of 7 mixed blocks as previously and 12 blocks (288 trials) produced entirely from memory. Additional testing that included trained and untrained sequences (consisting of untrained finger and interval orders) presented as instructed trials was also conducted before and after training blocks on day 1 and 2, respectively. These included trained and untrained sequences presented as instructed trials. The MEG session consisted of 2 mixed blocks (16 instructed and 32 from memory trials) for participants to refresh their memory of the sequences and become accustomed to the MEG environment. This was followed by 10 blocks with concurrent MEG and EMG recordings containing trials with sequences produced entirely from memory (giving a total of 240 trials split evenly between the four trained sequences).

#### MEG recordings

MEG was recorded continuously at 1200 samples/second using a whole-head 275-channel axial gradiometer system (CTF Omega, VSM MedTech) while participants sat upright in a magnetically shielded room. Head position coils were attached to nasion, left, and right auricular sites to provide anatomical co-registration.

#### EMG recordings

Participants were also fitted with four EMG electrodes to measure finger movement-related muscular activity. The electrodes were placed above the flexor carpi radialis (FCR), abductor polices brevis (APB), abductor digiti minimi (ADM), first dorsal interossei (FDI). FCR was recorded with a belly-belly montage, APB, ADM and FDI with a tendon-belly montage.

### Quantification and Statistical Analysis

#### Behavioral data analysis

To determine temporal accuracy in trials produced entirely from memory, we calculated a mean absolute deviation from target interval structure for each trial expressed as percentage of the target intervals. For the pre and post-test which consisted of instructed trials only, we calculated the absolute reaction time deviation from the finger cues to which the participant had to synchronize.

#### MEG and EMG preprocessing

MEG data analysis made use of SPM8 (Litvak et al., 2011, Wellcome Trust Centre for Neuroimaging, London, United Kingdom), Fieldtrip (Oostenveld et al., 2011) Donders Institute for Brain Cognition and Behavior) and custom MATLAB code. MEG data were downsampled to 1000 Hz, epoched for pre-processing into long trials spanning −2.8 to +12 s around the fractal cue to include a baseline fixation, fractal cue, ‘Go’ cue, sequence production and feedback. A 48-52Hz stopband filter was then applied to remove the 50 Hz power line noise within these long epochs. Channel artifacts were inspected in each participant, but no channels were identified as corrupted in any of the datasets. Due to the need to retain as many trials as possible for pattern classification and the involvement of long epochs, physiological artifacts related to heart rate, eyeblink, and breathing were identified based on the characteristic topography and time-course for each participant using ICA (RUNICA algorithm) and removed from the dataset. This procedure was carried out blind to the sequence conditions using the Fieldtrip component data browser, which allows the inspection of the topography and trial-by-trial time-course of the components. ICA-corrected data were then submitted to multivariate classification analysis, with the test probabilities calculated for a shorter epoch encompassing the preparation and production phases only (−2 to 4.5 s after the ‘Go’ cue). The EMG data were downsampled, epoched, and filtered in the same way as the MEG data. No trials were removed from the dataset, so that the EMG data from the same trials as in the MEG dataset was submitted to multivariate classification analysis.

#### Pattern classifier analysis

First we trained a standard LDA classifier to distinguish the MEG activity at the onset of each of the five button presses in a sequence. Following preprocessing, mean signal amplitude on each sensor during the 10ms period immediately before the onset of each response button press in the sequence was determined for all correct trials (mean proportion of correct trials across participants: 97.3% (SD: 3.1%); range: 87.2%–99.7%;). These mean signal amplitude values were used as a training dataset for all correct trials of each of the four sequences, respectively. The mean sensor pattern for each button press, and the common sensor-by-sensor co-variance matrix was determined from the training dataset. A Gaussian-linear multi-class classifier (*cf.* Supplementary Material) was then used to calculate the posterior probability of an activity pattern belonging to each of the five presses in the sequence across non-overlapping 10ms time windows in each trial of a) the same sequence (‘within’), b) a sequence with the same target timing, but a different finger order (‘temporal’), c) a sequence with the same finger order, but a different target timing (‘spatial’) and d) a sequence with both a different finger order and timing than the training sequence (‘positional’). The same procedure was used for the analysis of EMG patterns.

For statistical analysis of CQ during sequence preparation, an average probability for each of the five press patterns was determined for per trial in a 1 s time window immediately before the ‘Go’ cue. We then quantified the CQ strength in each trial by taking the median difference in posterior probabilities for each pair of consecutive finger presses in each trial (i.e., from 1^st^ to 2^nd^, 2^nd^ to 3^rd^, 3^rd^ to 4^th^ and 4^th^ to 5^th^), and then the mean distance across trials. Since the 1^st^ press had a special status with the finger identity and target onset timing remaining the same across sequences for each participant, we also calculate this distance measure without the 1^st^ press probability (median difference from 2^nd^ to 3^rd^, 3^rd^ to 4^th^ and 4^th^ to 5^th^). To display pattern probability dynamics, probabilities for each finger press pattern were averaged across trials for each 10ms time window (*cf.* Figure S1 for 5ms, 20ms and 50ms windows analyses), and then averaged across sequences. For display purposes only, the data were smoothed using a sliding average boxcar of 100 ms (i.e., ten 10 ms time bins) using the MATLAB fastsmooth function (https://uk.mathworks.com/matlabcentral/fileexchange/19998-fast-smoothing-function).

The ‘within’ sequence analysis trained and tested the classifier on time windows (training on production, testing on preparation data) from the same set of trials. Accordingly, the classification of production-related time-windows – i.e., 10ms before the 1^st^-5^th^ press, respectively – was performed on the training data, running the risk of over-fitting (although it is important to note that the specific timing of finger presses, and therefore the specific time bin from each trial used to train the classifier, varied from trial to trial). Nonetheless, to support the validity of press probability dynamics during the production period, we performed a 10-fold cross-validation ‘within’ sequence analysis, in which the classifier was successively trained on 90% of trials and then tested on the remaining 10% until all trials had formed part of the test dataset, with press probability dynamics averaged over all ten folds. Importantly, these press probability curves showed qualitatively the same phasic increases and decreases as in the original ‘within’ sequence analysis (Figure S3).

#### Source reconstruction

The linearly constrained minimum variance (LCMV) beamformer spatial filter algorithm from the Dynamic Analysis in Source Space (DAiSS) toolbox for SPM12 was used to estimate cortical activity on a 10mm grid for whole brain analyses based on preprocessed MEG data. Co-registration to MNI coordinates was based on nasion, left and right pre-auricular fiducial points, and the forward model was derived from a single shell fit to the inner skull surface of a canonical T1 image. There data were then submitted to searchlight analysis.

#### Searchlight analysis

To identify the neural origin of the CQ signal during sequence preparation at the sensor and the source levels we used a searchlight approach in combination with LDA. The searchlight size corresponded to ∼1% of the data, i.e., three sensors at the sensor level and 20 voxels at the source level. For the focal LDA analysis at the sensor level, we first determined the two nearest neighbors of each sensor based on the MEG sensor layout. As in the case of the whole-head analysis, the LDA classifier was then trained on mean signal amplitude from those three sensors during the 10ms period immediately before the onset of each physical button in correct trials. Next, we tested the classifier on mean signal amplitude from the same three sensors in a single 10ms time window 500ms before the ‘Go’ cue and quantified the probability of each finger press being decoded in each trial. Finally, as in the previous CQ distance analysis, we computed the median distance between the probability of the 1^st^ to 5^th^ (‘within’) finger presses, respectively, averaged across trials for each sequence and then across sequences for each participant. We then z-transformed the median distance values across sensors for each participant and computed a one-sample t-statistic on z-score values at each sensor across participants. There data were then plotted as t-statistic at the scalp level and significant sensors at p < 0.01 marked with a point and a label.

The same analysis was applied to mean signal amplitude in source space using searchlights of 20 voxels as features in the LDA. Whole brain results were subjected to a random effects analysis with an uncorrected threshold of *t*(15) > 3.73, p < 0.00. Peak voxels and t-values of significant clusters were then reported, and the data plotted as t-statistics with a threshold of *t*(15) > 5.24, p < 0.05 (FWE corrected at the voxel level) centered on the peak voxel (Worsley et al., 1996). Peak voxels and t-values of significant clusters were then reported in the results and the data plotted as a t-statistic at the scalp level at a threshold of *t*(15) > 5.24, p < 0.05 (FWE corrected at the voxel level) centered at the peak voxel (MNI coordinates: 30, −30, −24). The data thresholded at *t*(15) > 3.73, p < 0.001 (uncorrected) can be found in Figure S7A.

Finally, to identify regions that were driving the differences between finger presses during the sequence production period, we employed a searchlight approach in combination with LDA using data from the production period only. As in the analysis described above, the searchlight size corresponded to ∼1% of the data – three sensors at the sensor level and 20 voxels at the source level. Here the LDA classifier was trained to distinguish between the activity patterns of sensors or voxels during the 10ms period immediately before the onset of each physical button in correct trials. We used a five-fold cross-validation procedure (iteratively training on 80% of trials and testing on the remaining 20%) and quantified the probability of each finger press being correctly identified within trials for each finger press sequence. The data were than averaged across sequences and z-scored across sensors or voxels within each participant. Sensor accuracy results were then plotted as t-statistic at the scalp level and significant sensors at p < 0.01 marked with a point and a label. At the source level, whole brain results were corrected using a random effects analysis with an uncorrected threshold of t(15) > 3.73, p < 0.001. Peak voxels and t-values of significant clusters were then reported in the results and the data plotted as t-statistics at the scalp level with a threshold of *t*(15) > 5.93, p < 0.05 (FWE corrected at the voxel level) centered on the peak voxel (MNI coordinates: −22, −38, 52) (Worsley et al., 1996). The data thresholded at t(15) > 3.73, p < 0.001 (uncorrected) can be found in Figure S7B.

### Data and Software Availability

Upon publication MATLAB scripts for reproducing the multivariate classification analyses alongside one behavioral, MEG and EMG dataset of a representative participant, will be made available on Mendeley data (https://doi.org/10.17632/fxbmm66cr6.1). Upon publication group t-stat images of the source reconstruction will be made available on Mendeley data (https://doi.org/10.17632/fxbmm66cr6.1).

## Supplemental Information

Document S1. Figures S1–S7

Document S2. Article plus Supplemental Information
