# Supplementary material for: Neural Competitive Queuing of Ordinal Structure Underlies Skilled Sequential Action
Source: Neuron. 2019 Mar 20;101(6):1166–1180.e3. doi: 10.1016/j.neuron.2019.01.018 (PMC6436939; doi:10.1016/j.neuron.2019.01.018)
Supplement: Document S1. Figures S1–S7 [file mmc1.pdf]

Neuron, Volume *101*

Supplemental Information

Neural Competitive Queuing of Ordinal Structure Underlies Skilled Sequential Action

Katja Kornysheva, Daniel Bush, Sofie S. Meyer, Anna Sadnicka, Gareth Barnes, and Neil Burgess

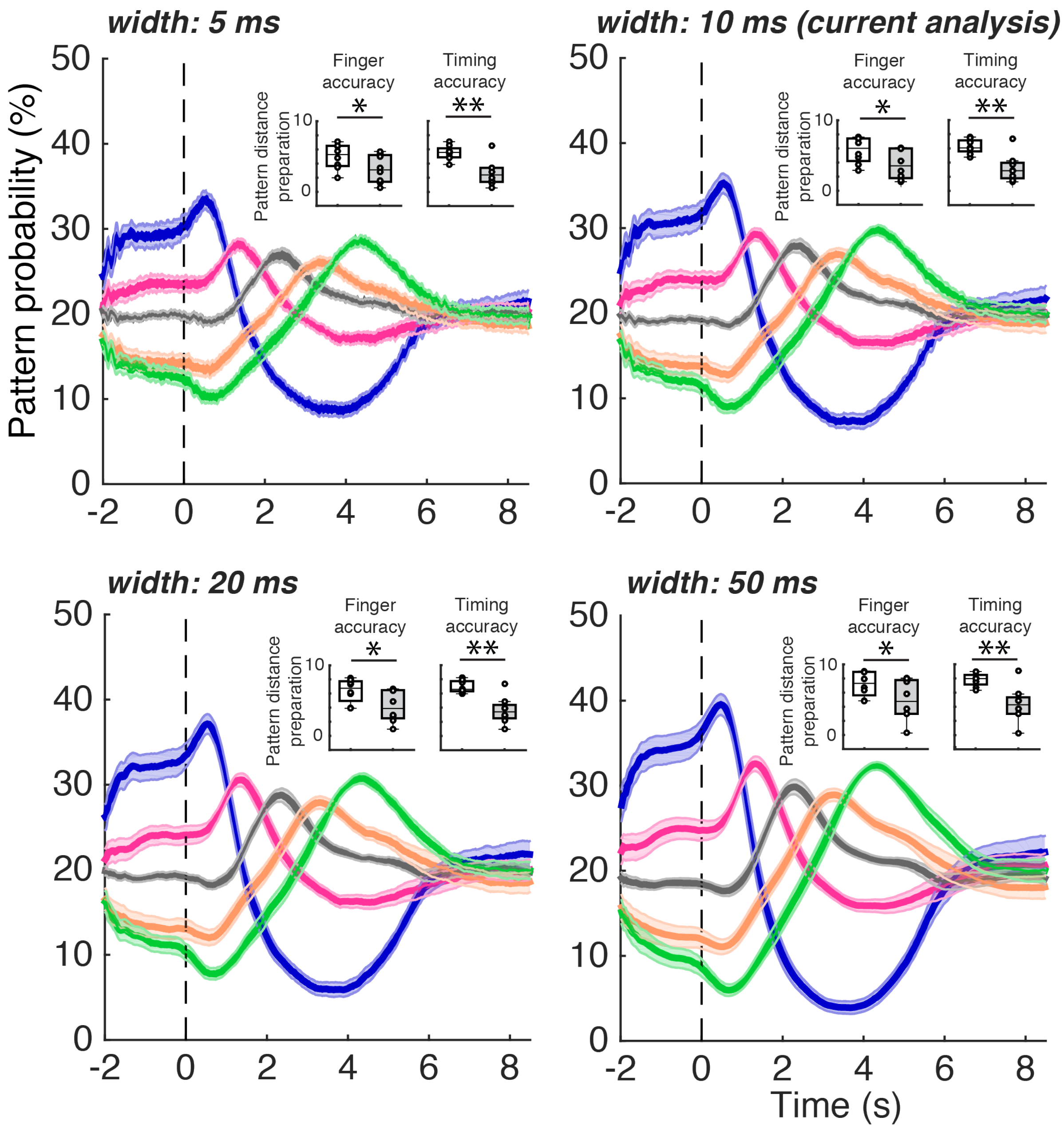

Figure S1. MEG pattern classification employing time windows of different width, Related to Fig. 5a, Fig. 6c and STAR Methods: The pattern probability curves show ordered competitive queuing during preparation and consistent associations with behavioural performance across classification analyses employing window width of 5, 10, 20 and 50 ms ('within' sequence classification).

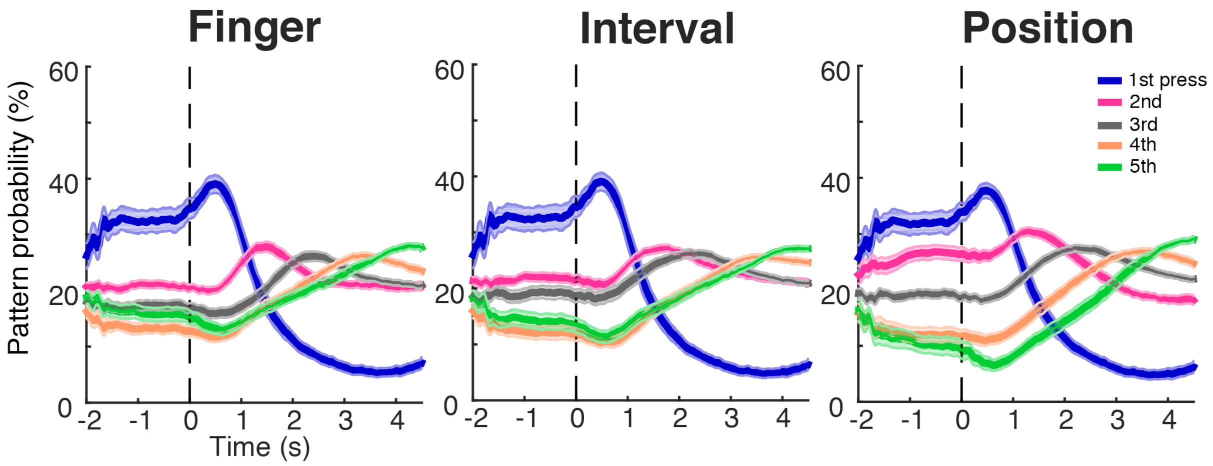

Figure S2. Probability values based on training the classifier on finger identity, interval identity and position across all sequences, Related to Fig. 5a: The classifier was trained on MEG data prior to presses with specific finger identities (collapsed across ordinal positions and different temporal intervals preceding the press), temporal intervals (collapsed across different ordinal positions and effected using different fingers), or ordinal positions (collapsed across different fingers and different temporal intervals preceding the press) across all sequences. The results are displayed according to the position of the finger press or preceding temporal interval in that sequence. Competitive queuing was most pronounced when training on specific ordinal positions (position versus finger 2nd-5th: $t(15) = 3.1458$, $p = .013$; positional versus interval 2nd-5th: $t(15) = 4.1165$, $p = 0.002$, two-sample t-test, Bonferroni-corrected for two comparisons).

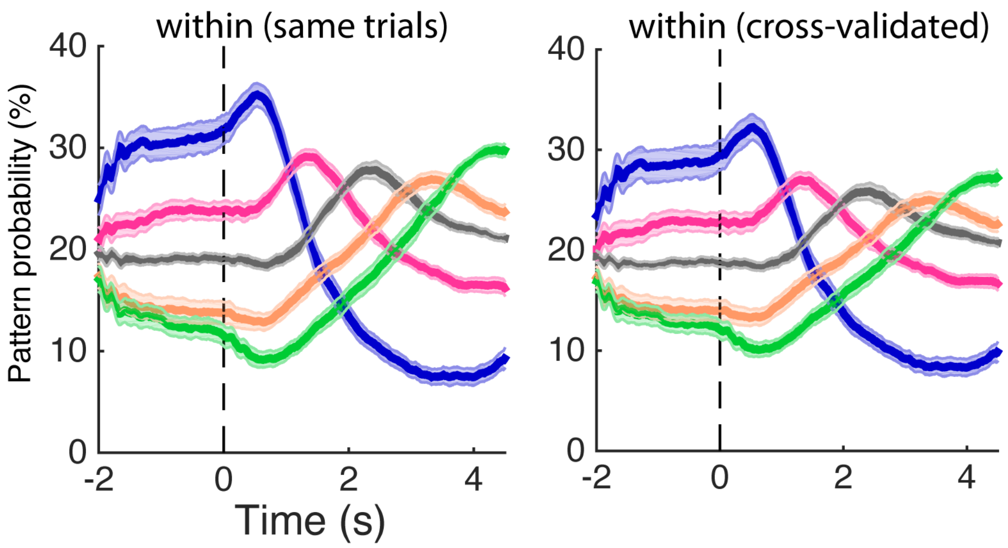

Figure S3. Cross-validated 'within' sequence analysis, Related to Fig. 5a: Press pattern probability dynamics for 'within' sequence analysis when testing across time-windows in the same and across different trials (10-fold cross-validation).

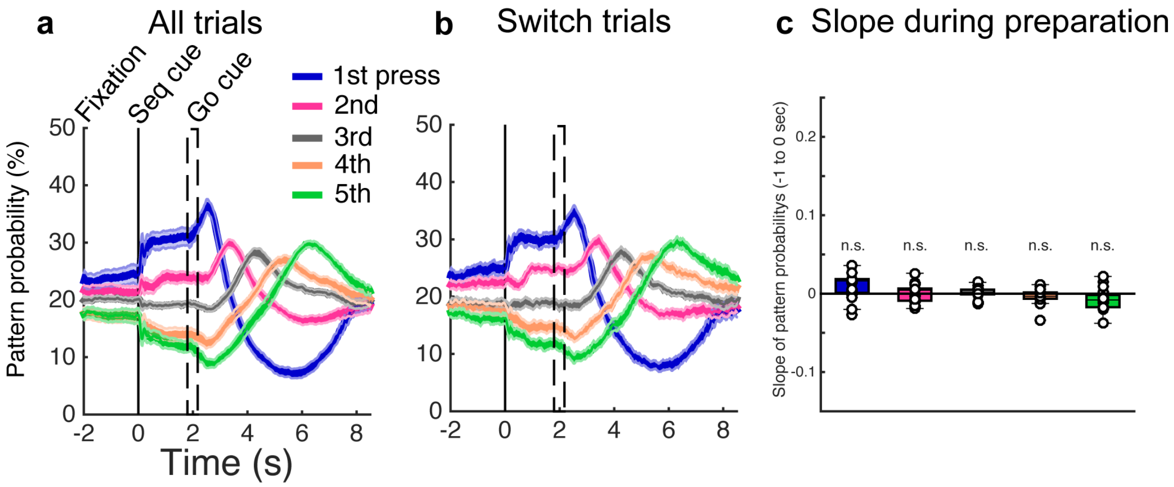

Figure S4. **Control analyses to test the alternative hypothesis that pattern probability during preparation reflects neural pattern proximity between training and testing time windows in the context of a slow-changing brain state, rather than the ordered queuing of sequential press patterns, Related to Fig. 5: a.** Pattern probabilities before and after the Sequence cue reveal a step change in the distance when analyzing all and **b.** only switch trials (preceding sequence different from current) **c.** No positive slope could be found for any of the press probabilities during the final 1 sec before the ‘Go’ cue.

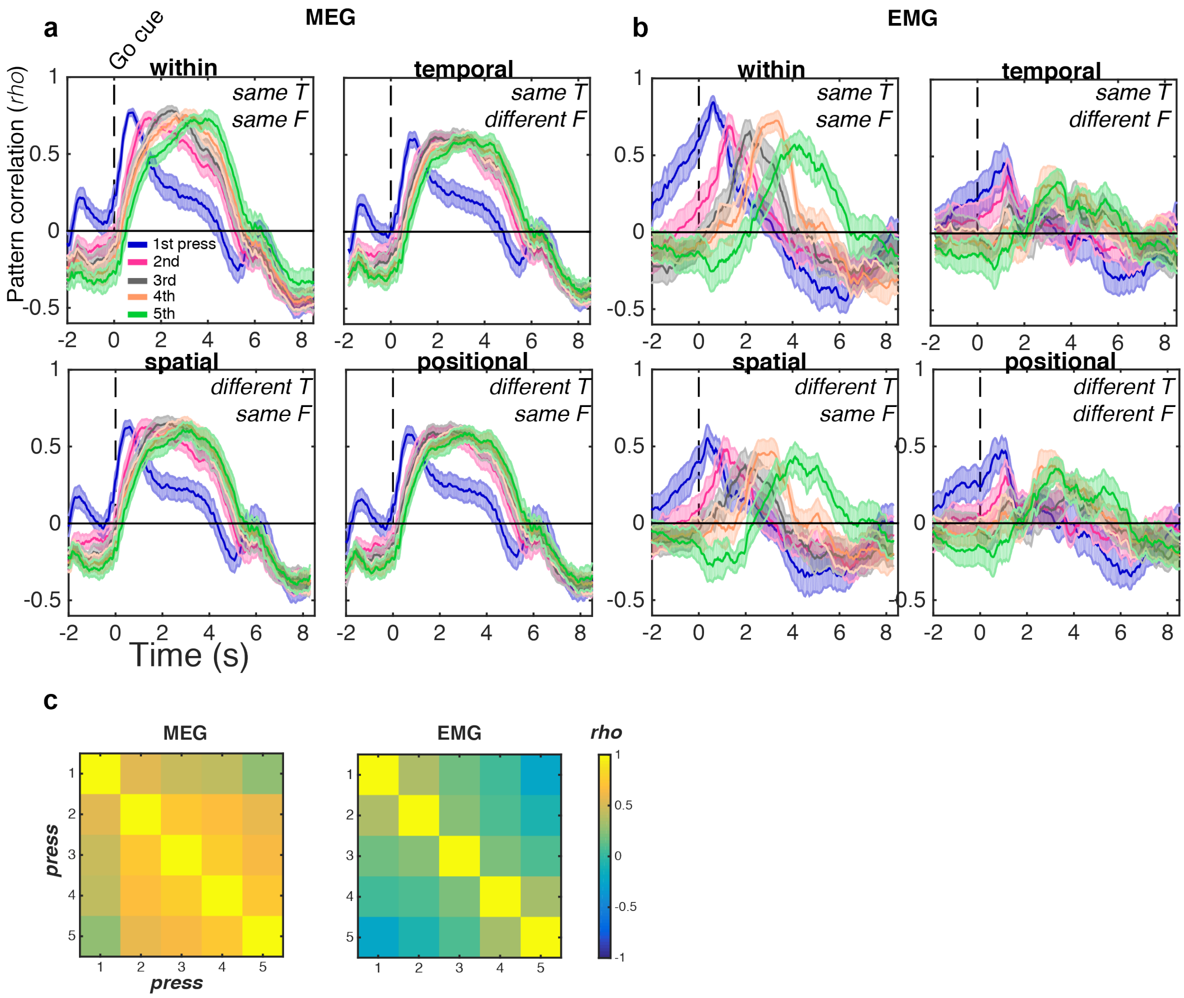

Figure S5. Pattern similarity across time windows, Related to Fig. 5a and c:
a. Dynamic correlation coefficients (*rho*) between MEG patterns for the 1st – 5th press (training patterns in the classification analyses) and time windows throughout the preparation and production periods. Pattern correlations peak during production time, similarly to pattern probabilities in Fig. 5, suggesting that our classification reflected increased pattern similarities. Note the presence of graded negative correlations during preparation for 2nd-5th press probabilities and the abrupt switch to positive correlations after the 'Go' cue, indicating abrupt rather than gradual pattern dynamics. **b.** Same analyses for EMG patterns, respectively. **c.** Pattern correlation between press patterns during production revealed a graded positive correlation for EMG, but not for EMG data.

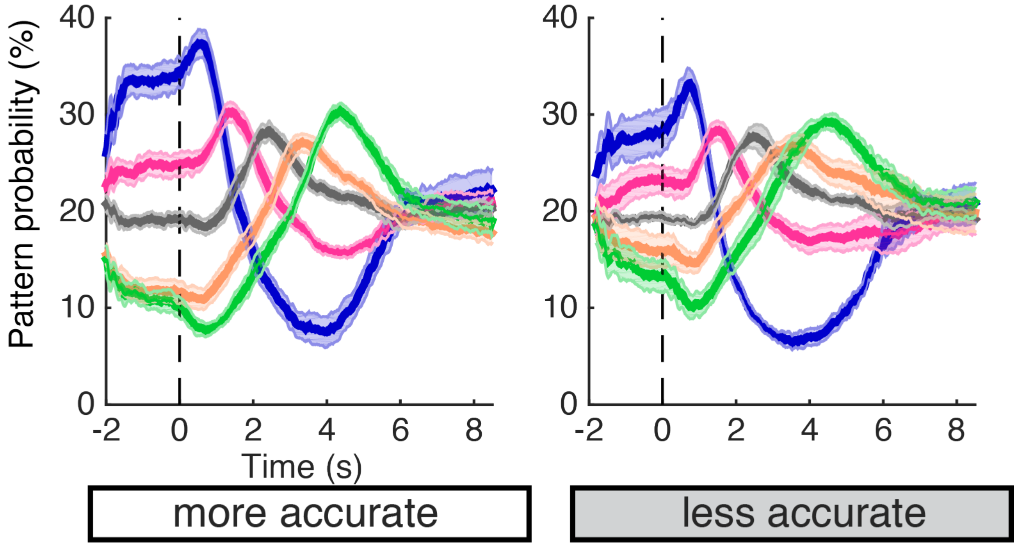

Figure S6. Median split by finger order accuracy, Related to Fig. 6a: Median split of press probability curves by percentage of trials with incorrect finger presses.

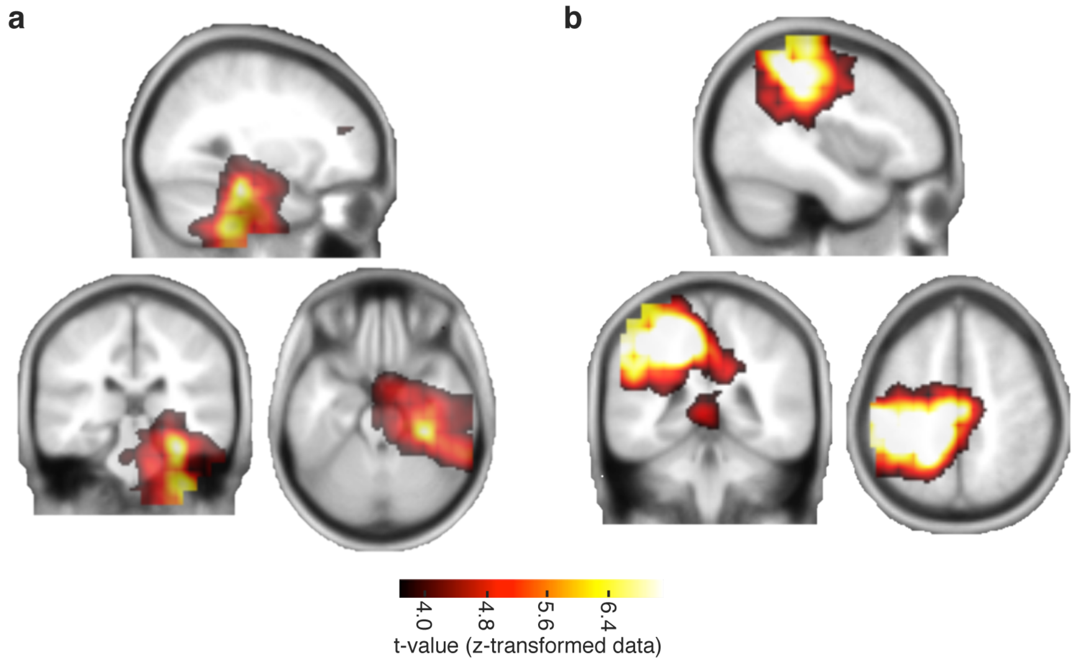

Figure S7. Source reconstruction, Related to Fig. 7c and d: The source reconstruction data as in Figure 7c-d plotted at a lower threshold of $t(15) > 3.73$, $p < .001$ (uncorrected) and centered at the respective peak voxel (MNI coordinates: 30, -30, -24 in panel **a**; -22, -36, 52 in panel **b**).
